# Supplementary material for: CoQ deficiency causes disruption of mitochondrial sulfide oxidation, a new pathomechanism associated with this syndrome
Source: EMBO Mol Med. 2016 Nov 17;9(1):78–95. doi: 10.15252/emmm.201606345 (PMC5210161; doi:10.15252/emmm.201606345)
Supplement: Supplementary file 1 — Appendix [file EMMM-9-78-s001.pdf]

Appendix table of content

Page 1: Appendix Figure Legends
Page 2: Appendix Figure S1
Page 3: Appendix Figure S2
Page 4: Appendix Figure S3
Page 5: Appendix Figure S4
Page 6: Appendix table S1

Appendix Figure Legends

Appendix Figure S1. CoQ biosynthetic pathway. Purple asterisk indicates the dysfunctional protein in the mouse models; orange asterisks indicate the dysfunctional proteins in the patients' skin fibroblasts. COQ9 is needed for the reaction catalyzed by COQ7. PDSS2 and COQ2 are catalytic enzymes. COQ4 may play a structural role in the multiprotein complex for CoQ biosynthesis.

Appendix Figure S2. **Glutathione system in muscle and kidney of** $\boldsymbol{Coq9^{R239X}}$ **mice.** Total GSH in cytosol and mitochondria of muscle *(A)* and kidney *(B)* of $Coq9^{+/+}$ and $Coq9^{R239X}$ mice. Cytosolic GPx and GRd activities in muscle *(C)* and kidney *(D)* of $Coq9^{+/+}$ and $Coq9^{R239X}$ mice. Data are expressed as mean ± SD. $*P < 0.05$; $Coq9^{R239X}$ mice versus $Coq9^{+/+}$ mice (*t* test; n = 5 for each group).

Appendix Figure S3. **Glutathione redox status in cerebrum, muscle and kidneys of** $\boldsymbol{Coq9^{R239X}}$ **mice.** GSSG / GSH ratio in cytosol (A) and mitochondria (B) from cerebrum, muscle and kidneys of $Coq9_{+/+}$ and $Coq9^{R239X}$ mice (*t* test; n = 5 for each group).

Appendix Figure S4. **Consequences of SQR deficiency in cellular GSH levels.** Levels of Sqr mRNA (*A*) SQR protein (*B*) and GSH (*C*) in Hepa1c1c7 cells after culture of SQR siRNA during 48 and/or 72 h. Levels of GSH (*D*) in fibroblasts of controls (C1-C3) and patients (P1-4) with primary CoQ_{10} deficiency. Data are expressed as mean ± SD. $***P < 0.001$; SQR siRNA versus negative siRNA control (*t* test or one-way ANOVA with a Tukey's *post hoc* test; n = 4 for each group).

<u>Appendix Figure S1</u>

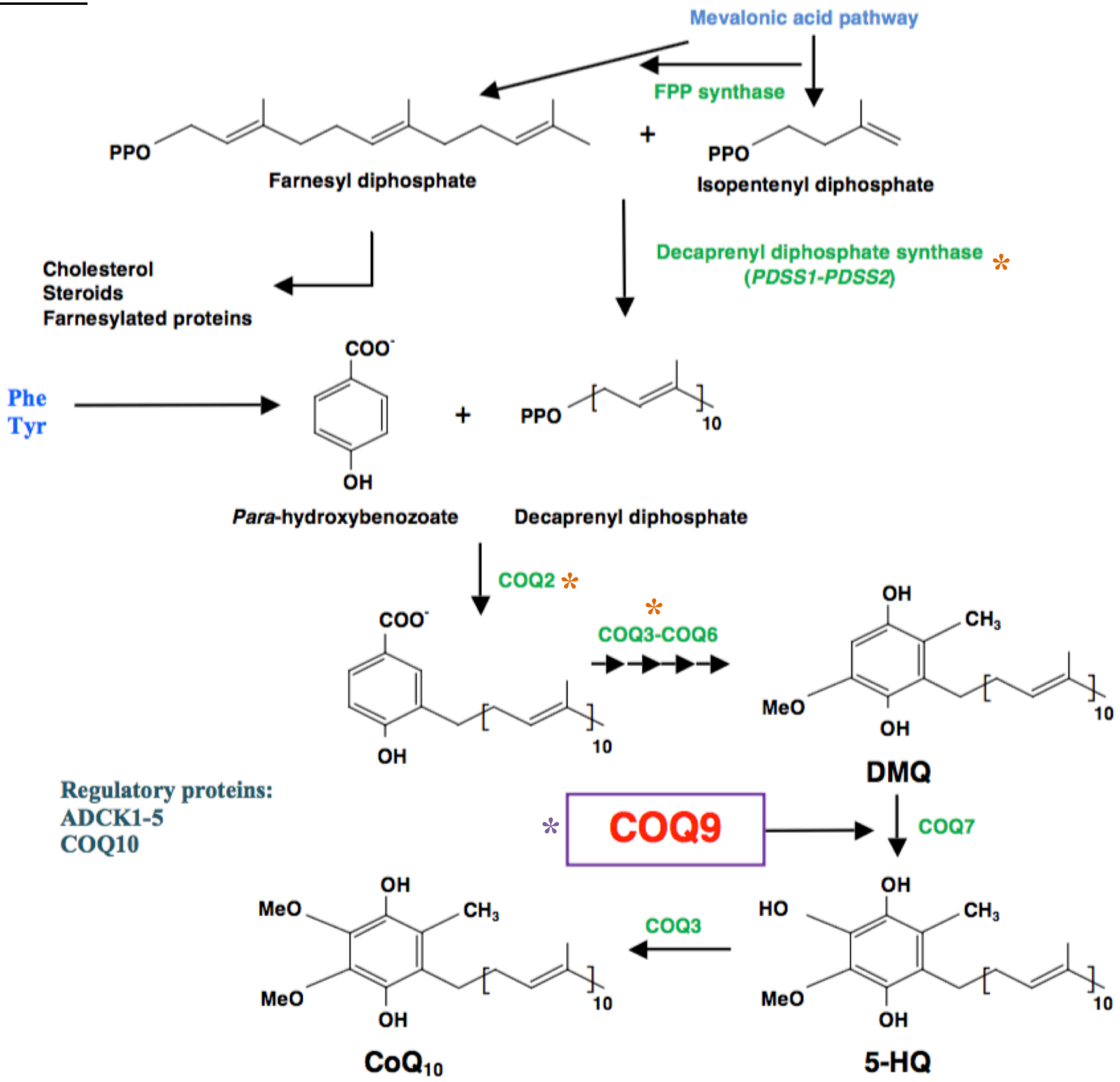

Appendix Figure S2

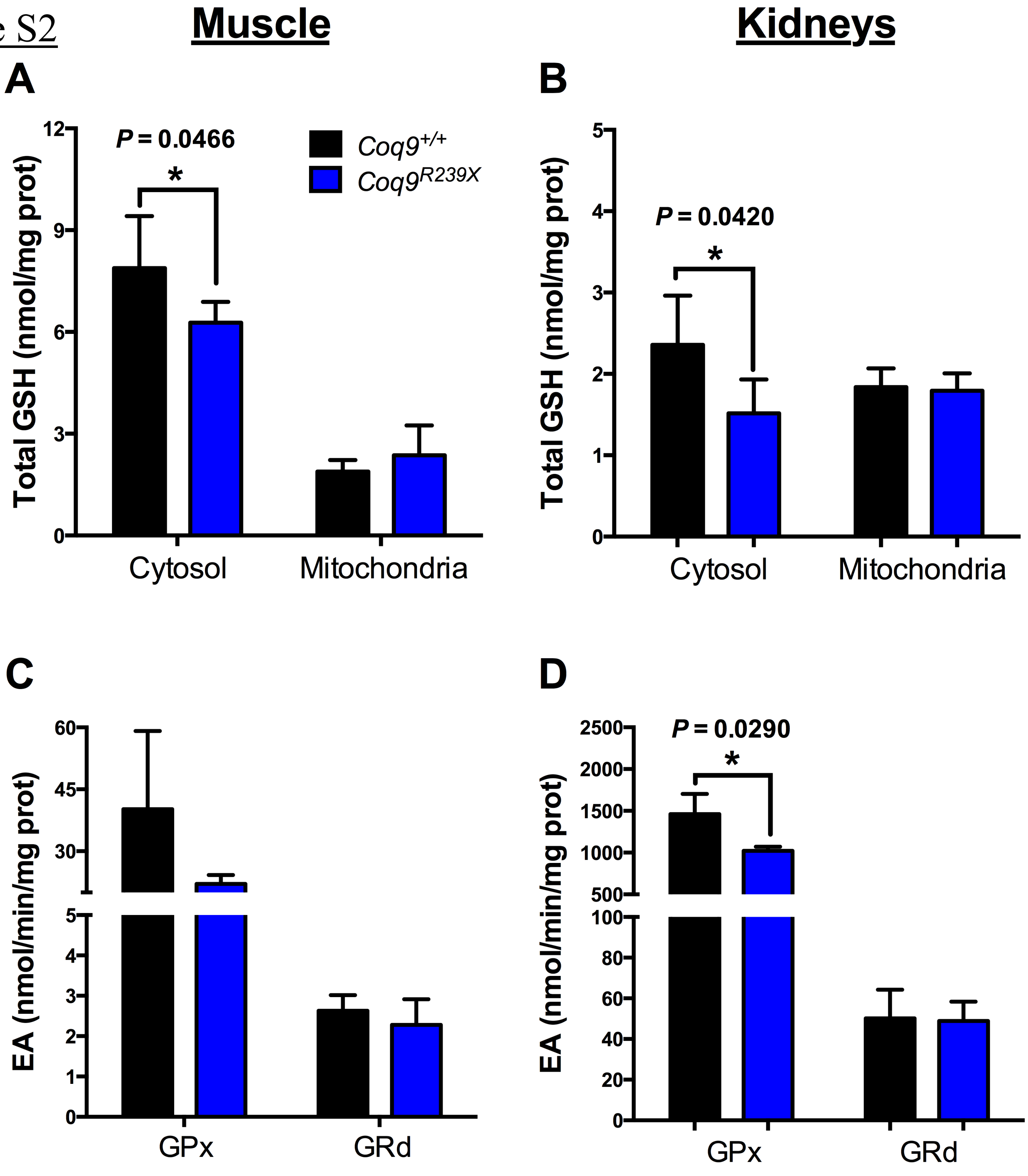

Appendix Figure S3

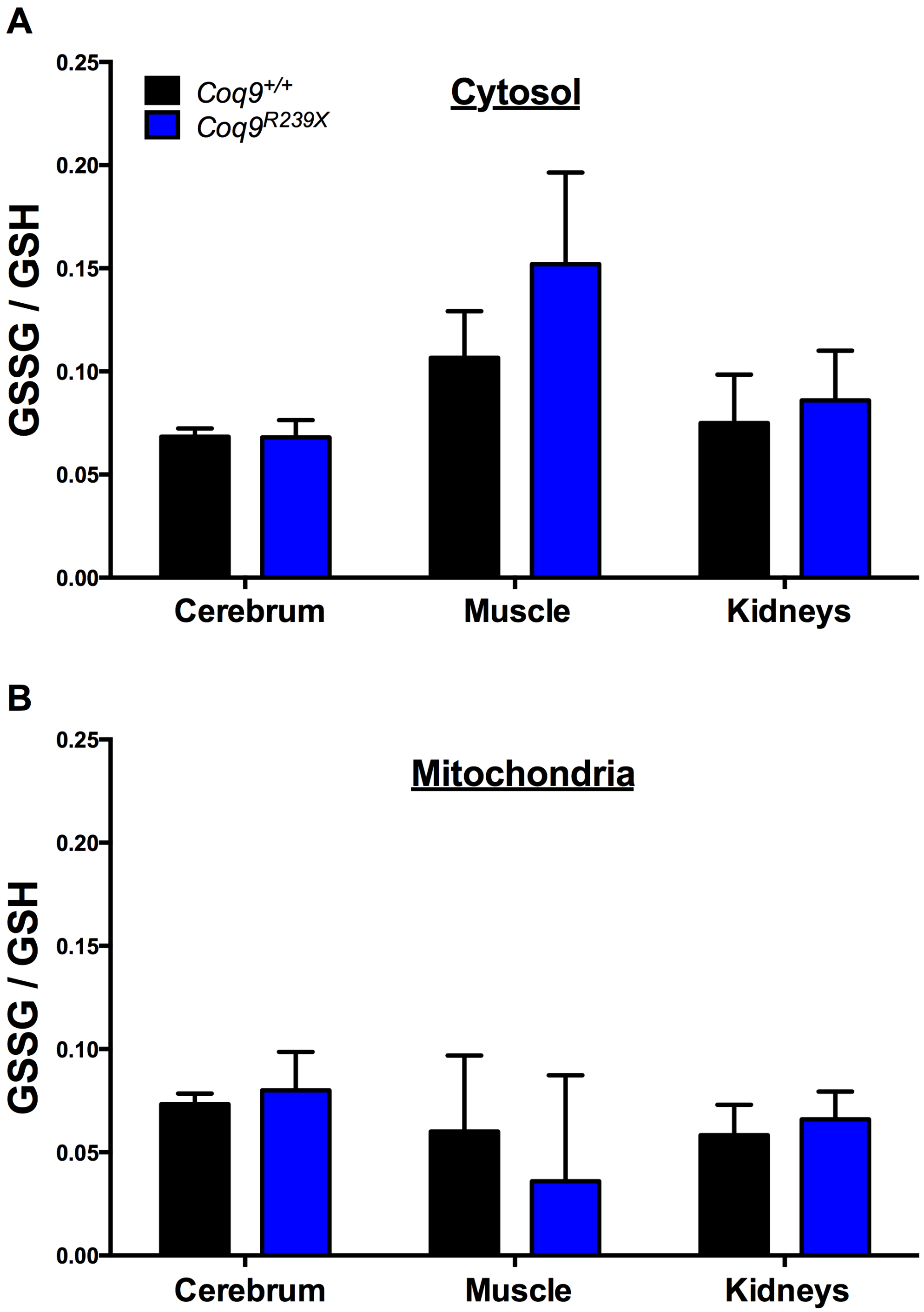

Appendix Figure S4

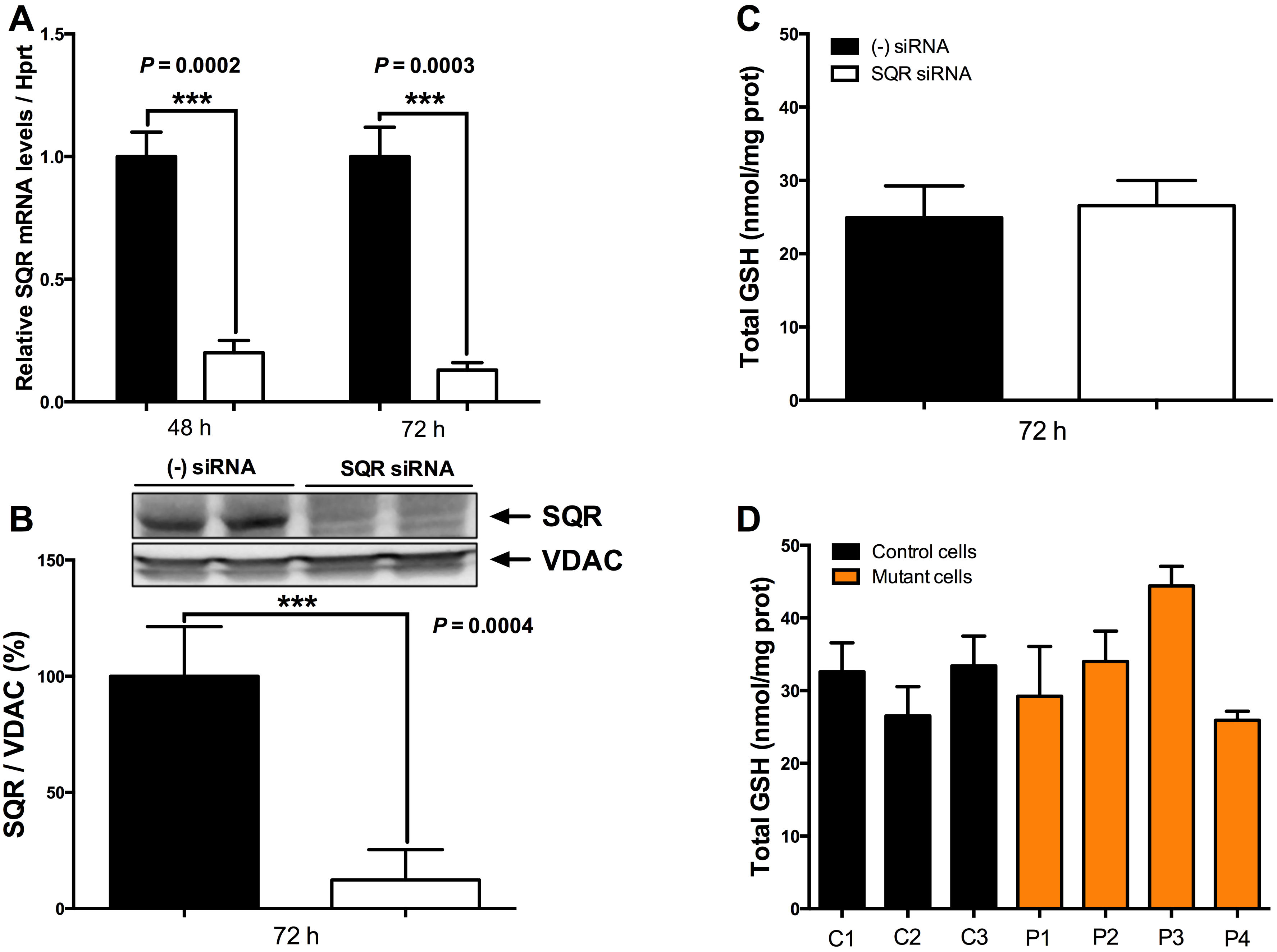

<u>Appendix Table S1</u>

Sample	Gene	Sequence Ref	Mutations	Protein change	Reference
P1	*PDSS2*	NM_020381.3	c.[964C>T];[1145C>T]	p.[Gln332Stop];[Ser238Leu]	López et al., 2006
P2	*COQ2*	NM_015697.7	c.760T>A	p.Trp254Arg	
P3	*COQ4*	NM_016035.3	c.[155T>C];[518_520delCCA]	p.[Leu52Ser];[Thr174del]	Brea-Calvo et al., 2015
P4	*COQ9*	NM_020312.3	c.521+1delG		Danhauser et al., 2015

Table S1. Identified mutations in the human skin fibroblasts used in this study.
