# Supplementary material for: CoQ deficiency causes disruption of mitochondrial sulfide oxidation, a new pathomechanism associated with this syndrome
Source: EMBO Mol Med. 2016 Nov 17;9(1):78–95. doi: 10.15252/emmm.201606345 (PMC5210161; doi:10.15252/emmm.201606345)
Supplement: Supplementary file 2 — Source Data for Appendix [file EMMM-9-78-s002.zip › EMM_06345_FigS4_source_data/EMM_06345_FigS4_source_data.pdf]

Figure S4. WB of SQR after *Sqr* silencing in Hepa cells.

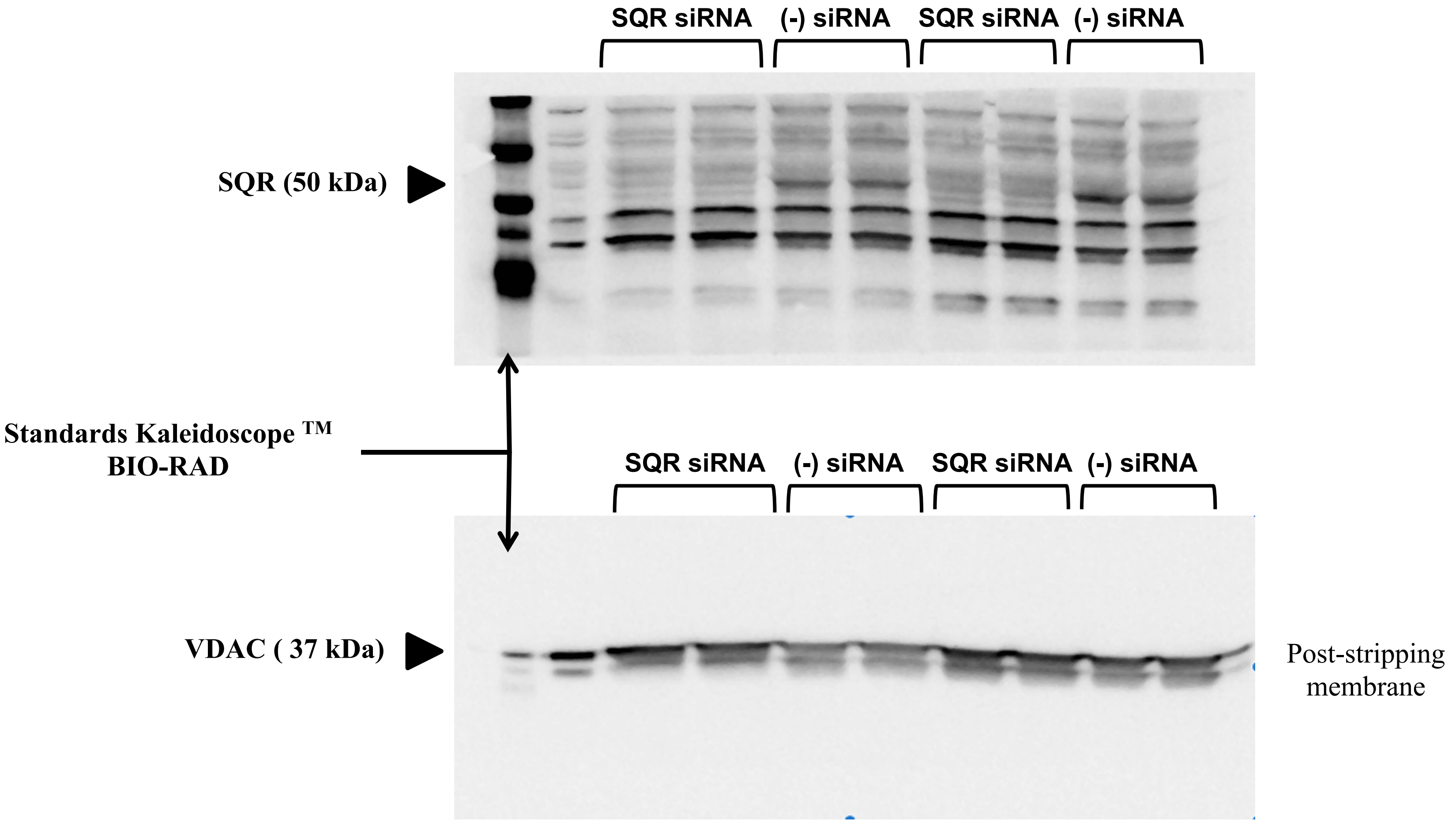

Note: lines 5, 6, 7 and 8 are represented in Figure S4 in the main text.
