# Supplementary material for: CoQ deficiency causes disruption of mitochondrial sulfide oxidation, a new pathomechanism associated with this syndrome
Source: EMBO Mol Med. 2016 Nov 17;9(1):78–95. doi: 10.15252/emmm.201606345 (PMC5210161; doi:10.15252/emmm.201606345)
Supplement: Supplementary file 4 — Source Data for Figure 2 [file EMMM-9-78-s003.pdf]

Figure 2D. SQR in cerebral mitochondria of wild-type and mutant mice.

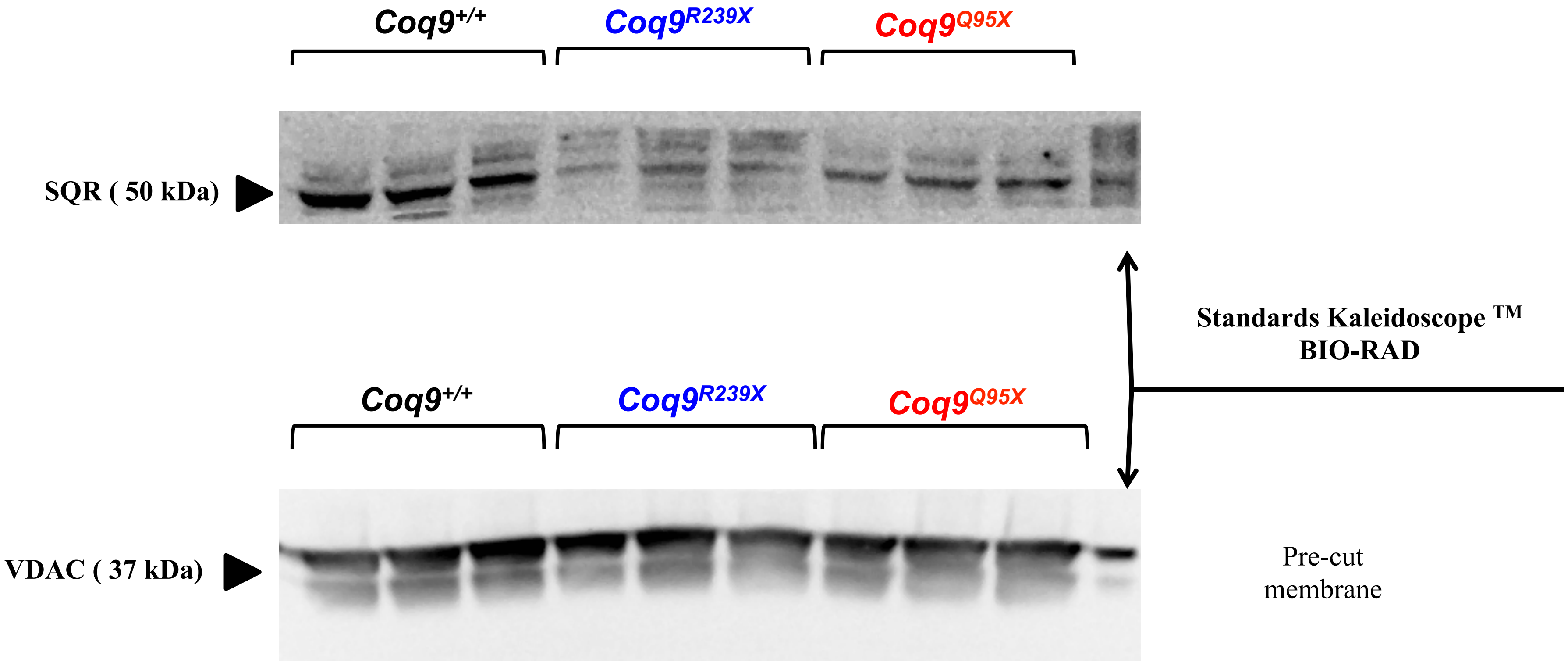

Note: lines 1, 2, 4, 5, 8 and 9 are represented in Figure 2D in the main text.

Figure 2E. SQR in kidneys of wild-type and mutant mice.

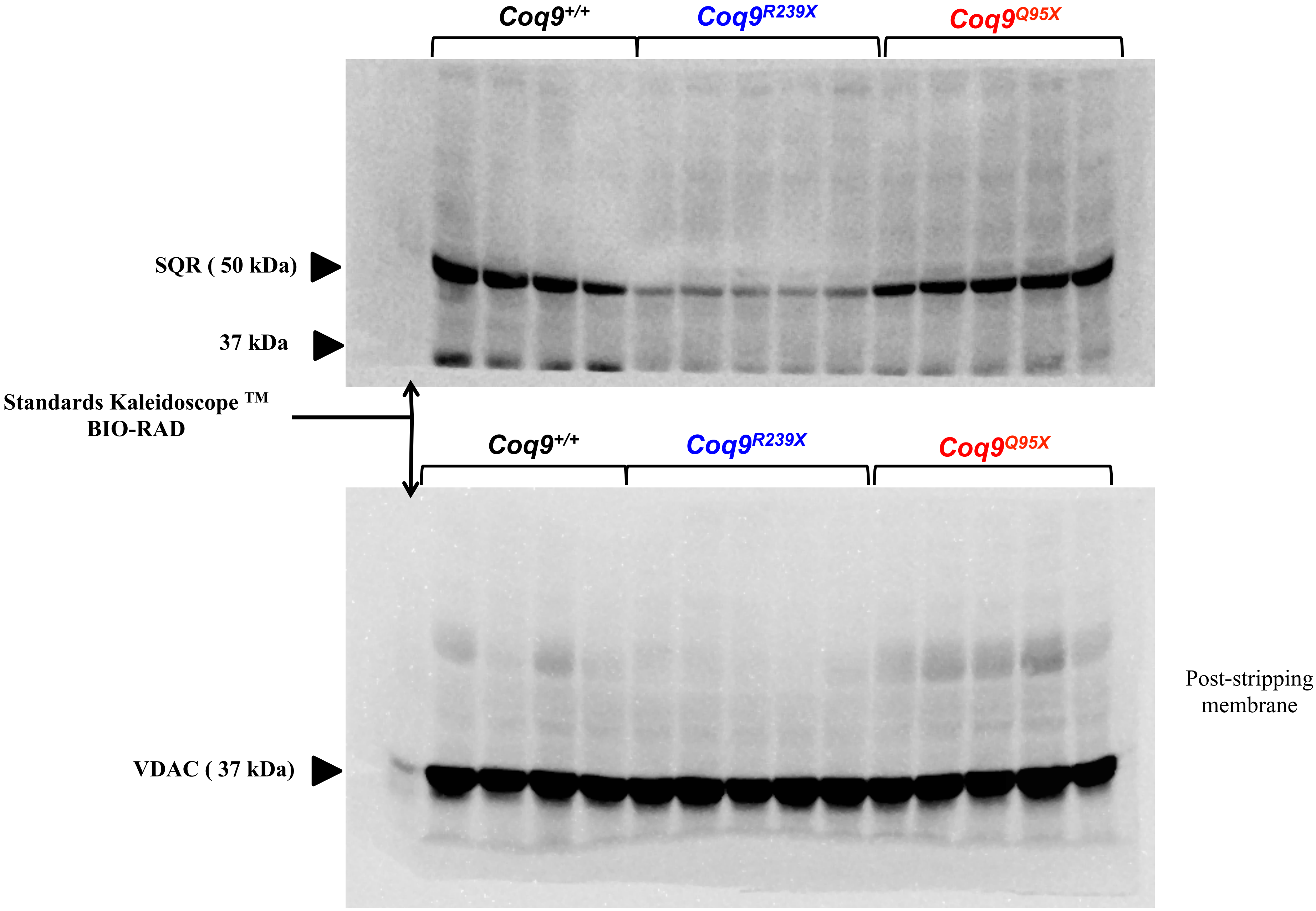

Note: lines 3, 4, 5, 6, 10 and 11 are represented in Figure 2E in the main text.

Figure 2F. SQR in muscle of wild-type and mutant mice.

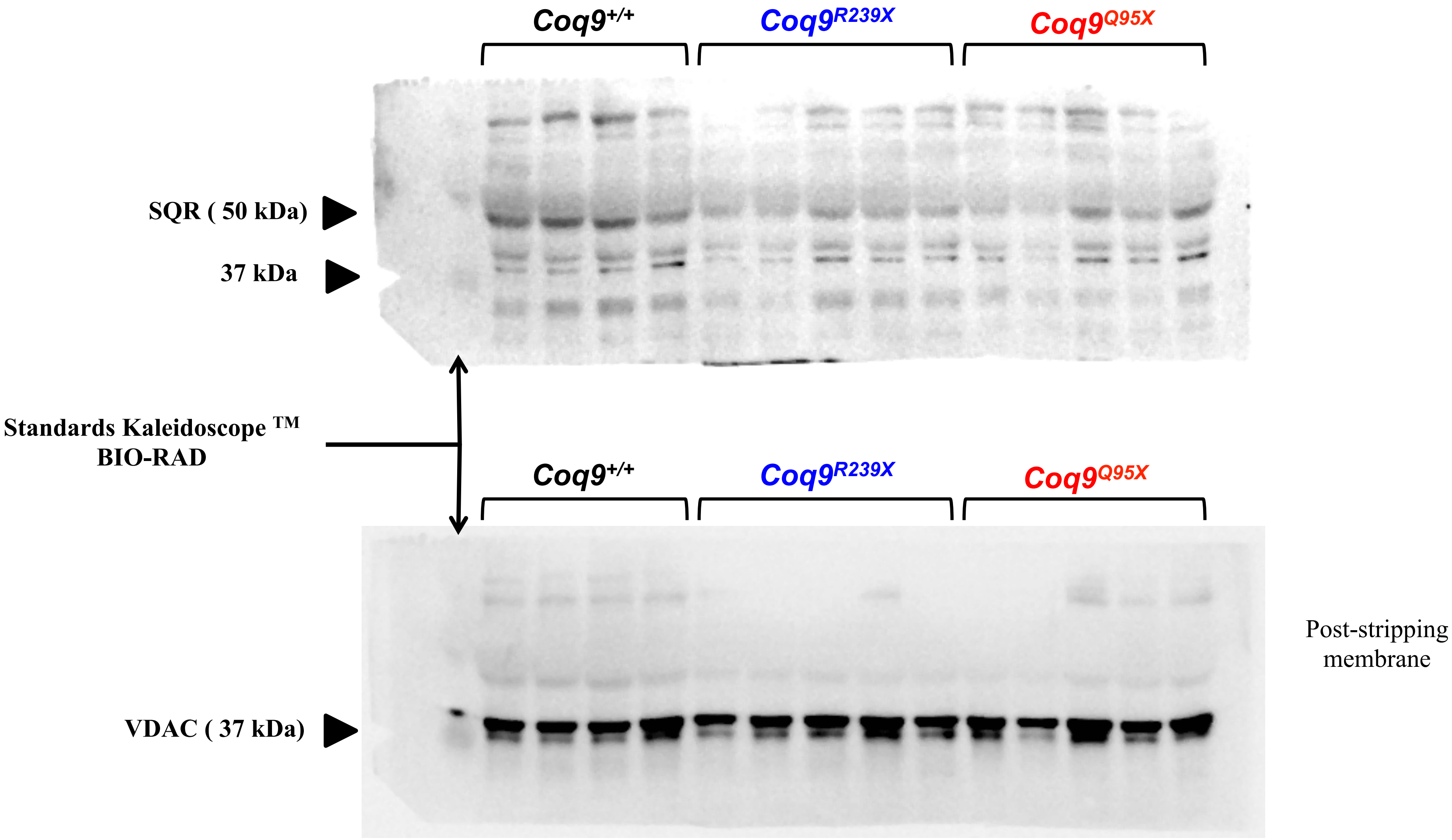

Note: lines 2, 3, 7, 8, 12 and 13 are represented in Figure 2F in the main text.
