# Supplementary material for: CoQ deficiency causes disruption of mitochondrial sulfide oxidation, a new pathomechanism associated with this syndrome
Source: EMBO Mol Med. 2016 Nov 17;9(1):78–95. doi: 10.15252/emmm.201606345 (PMC5210161; doi:10.15252/emmm.201606345)
Supplement: Supplementary file 5 — Source Data for Figure 3 [file EMMM-9-78-s004.pdf]

Figure 3B. SQR in human fibroblasts.

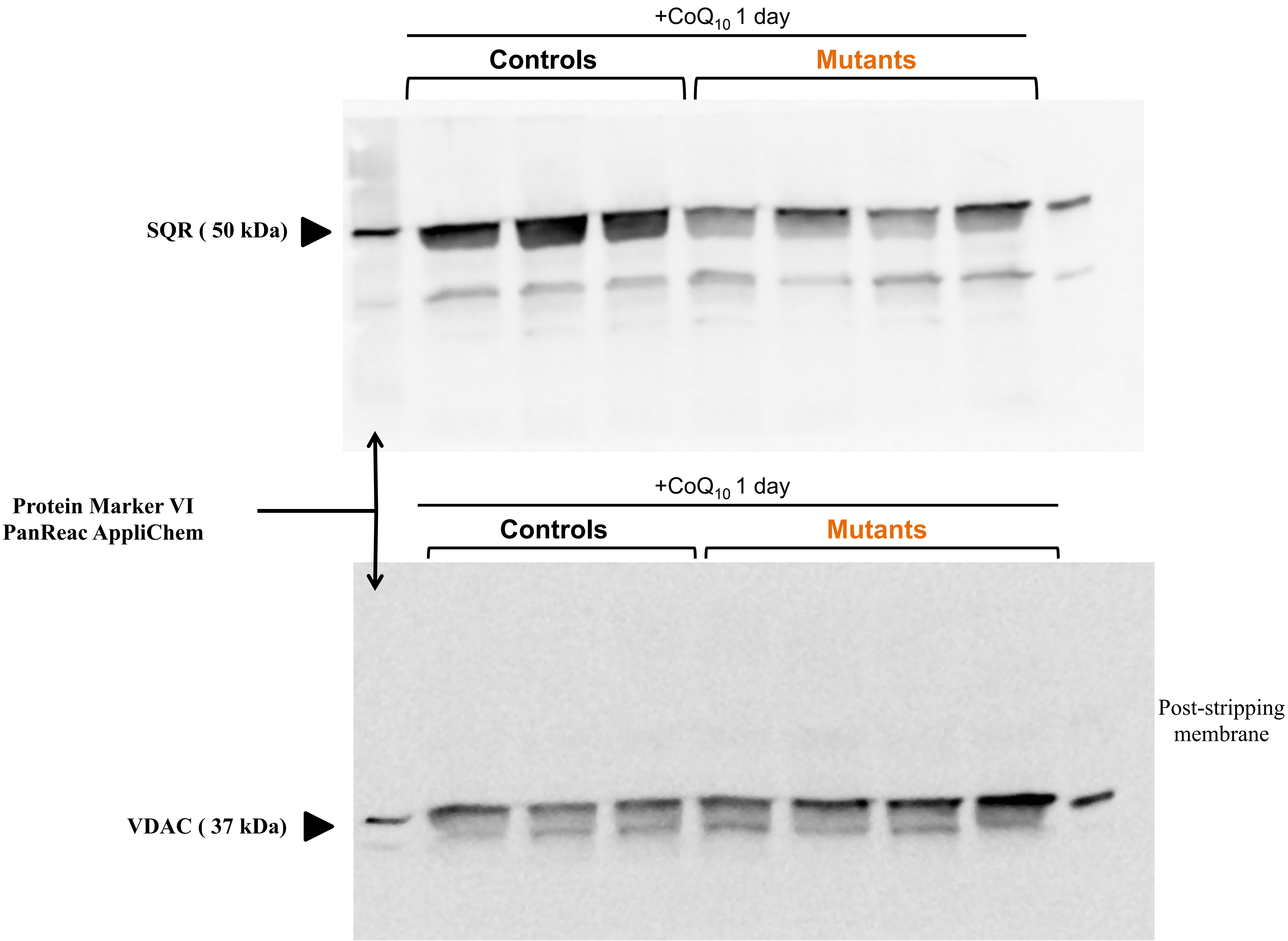

Figure 3E. SQR in kidneys of *Coq9^R239X^* after ubiquinol-10 supplementation.

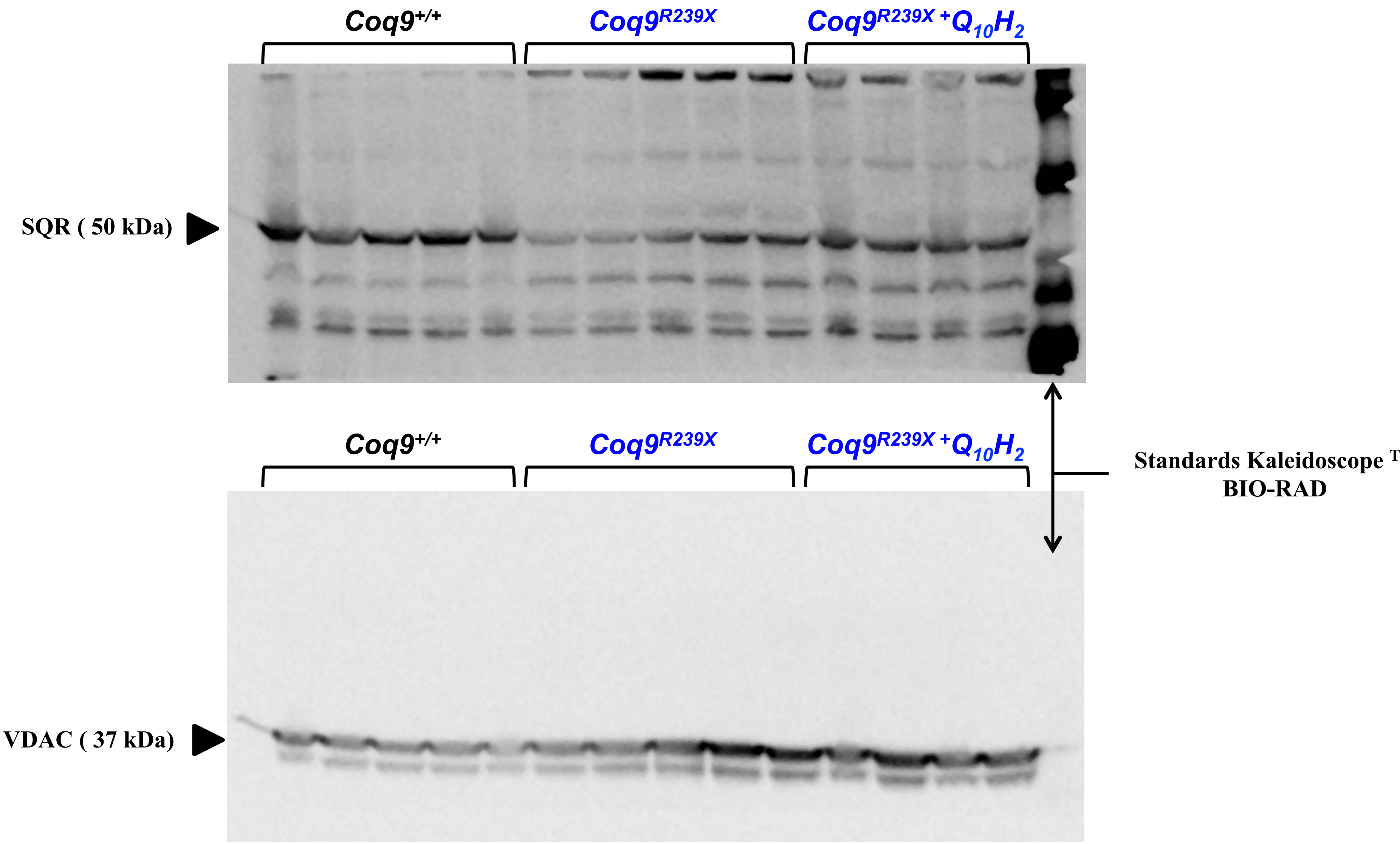

Note: lines 1, 2, 7, 8, 13 and 14 are represented in Figure 3E in the main text.

Figure 3F. SQR in muscle of *Coq9^{R239X}* after ubiquinol-10 supplementation.

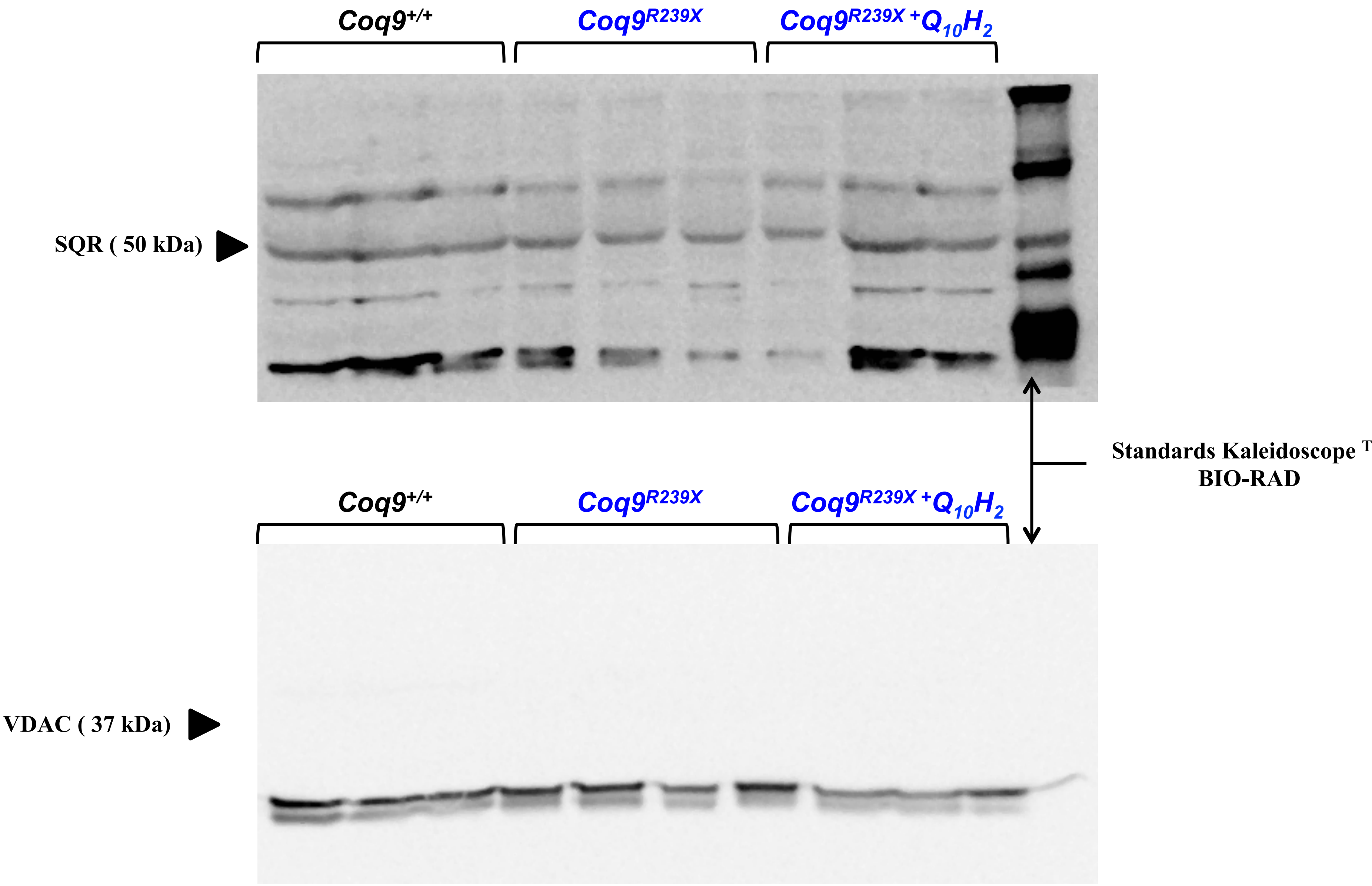

Note: lines 1, 2, 5, 6, 8 and 9 are represented in Figure 3F in the main text.
