# Supplementary material for: CoQ deficiency causes disruption of mitochondrial sulfide oxidation, a new pathomechanism associated with this syndrome
Source: EMBO Mol Med. 2016 Nov 17;9(1):78–95. doi: 10.15252/emmm.201606345 (PMC5210161; doi:10.15252/emmm.201606345)
Supplement: Supplementary file 6 — Source Data for Figure 4 [file EMMM-9-78-s005.pdf]

Figure 4A. TST1 in cerebrum of wild-type and mutant mice.

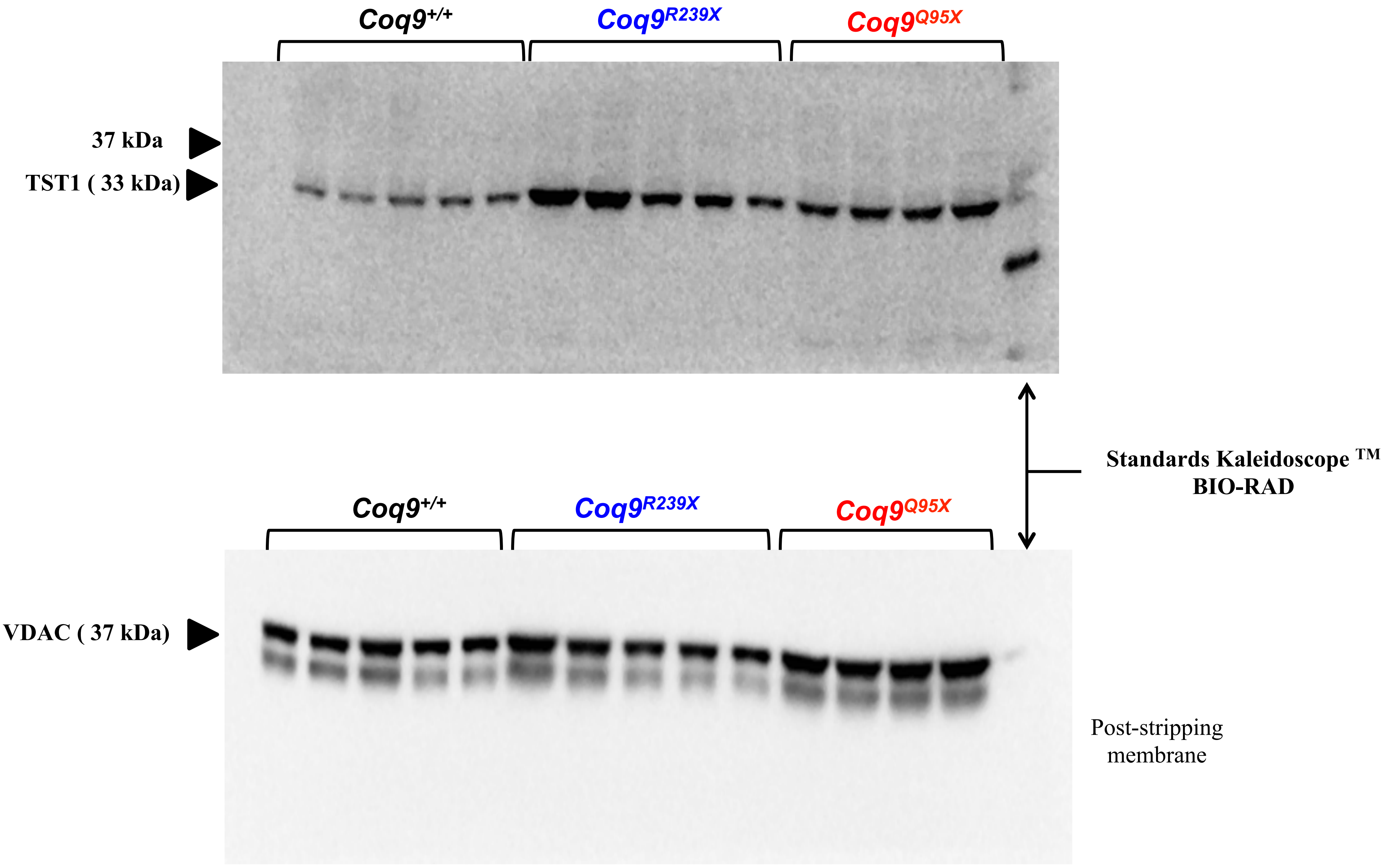

Note: lines 3, 4, 6, 7, 13 and 14 are represented in Figure 4A in the main text.

Figure 4B. TST1 in kidneys of wild-type and mutant mice.

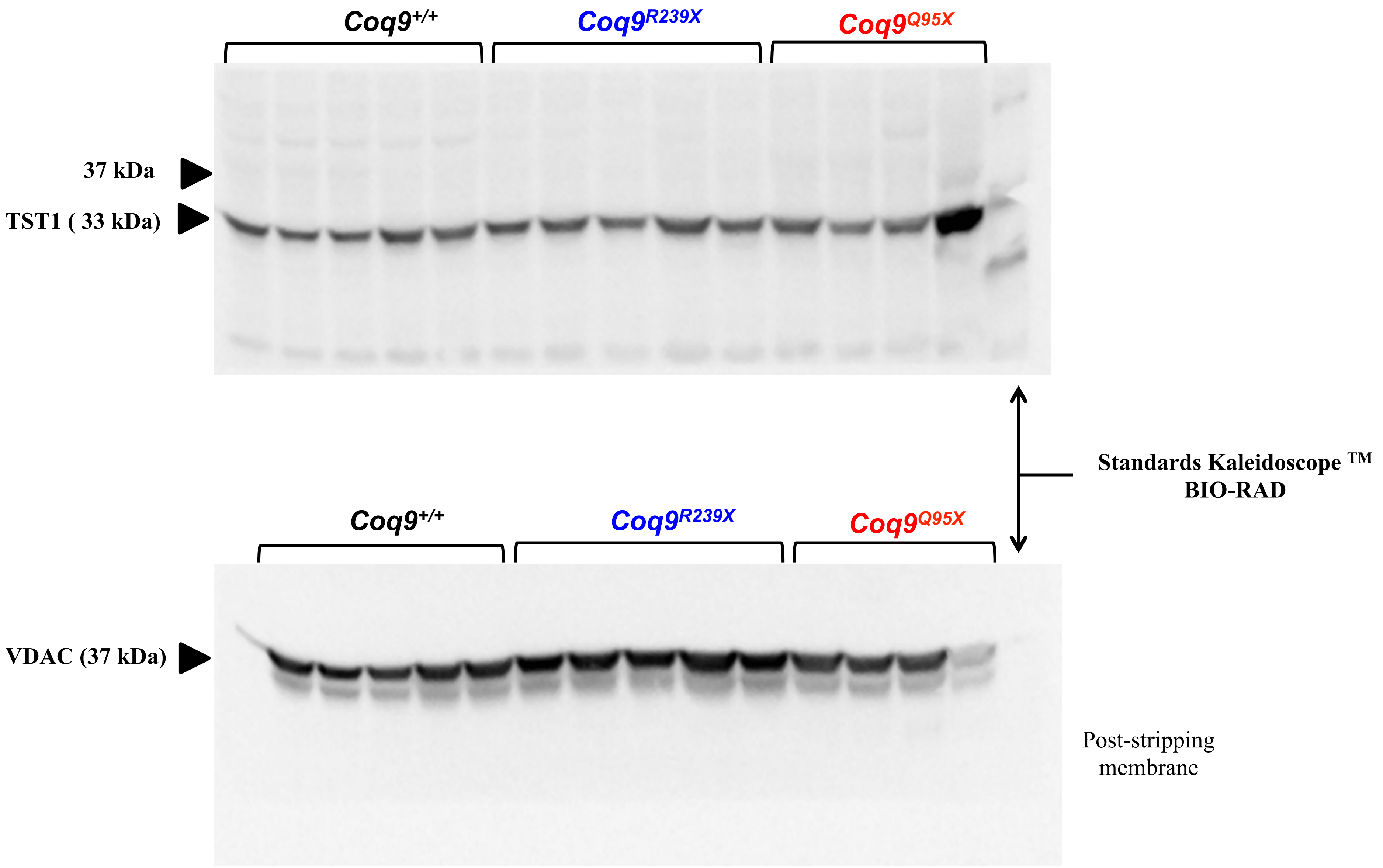

Note: lines 3, 4, 6, 7, 11 and 12 are represented in Figure 4B in the main text.

Figure 4C. TST1 in skeletal muscle of wild-type and mutant mice.

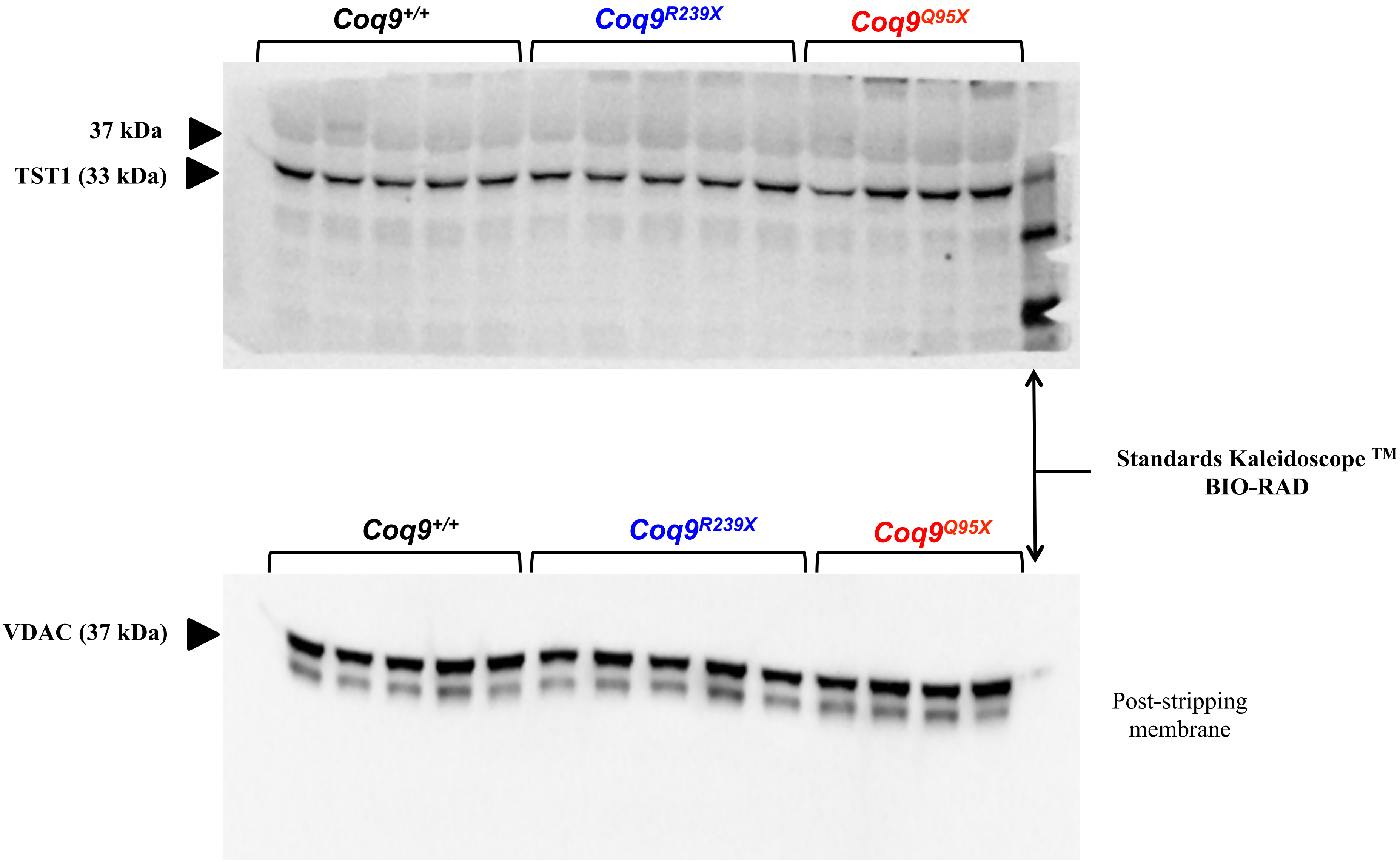

Note: lines 3, 4, 9, 10, 11 and 12 are represented in Figure 4C in the main text.

Figure 4G. ETHE1 in cerebrum of wild-type and mutant mice.

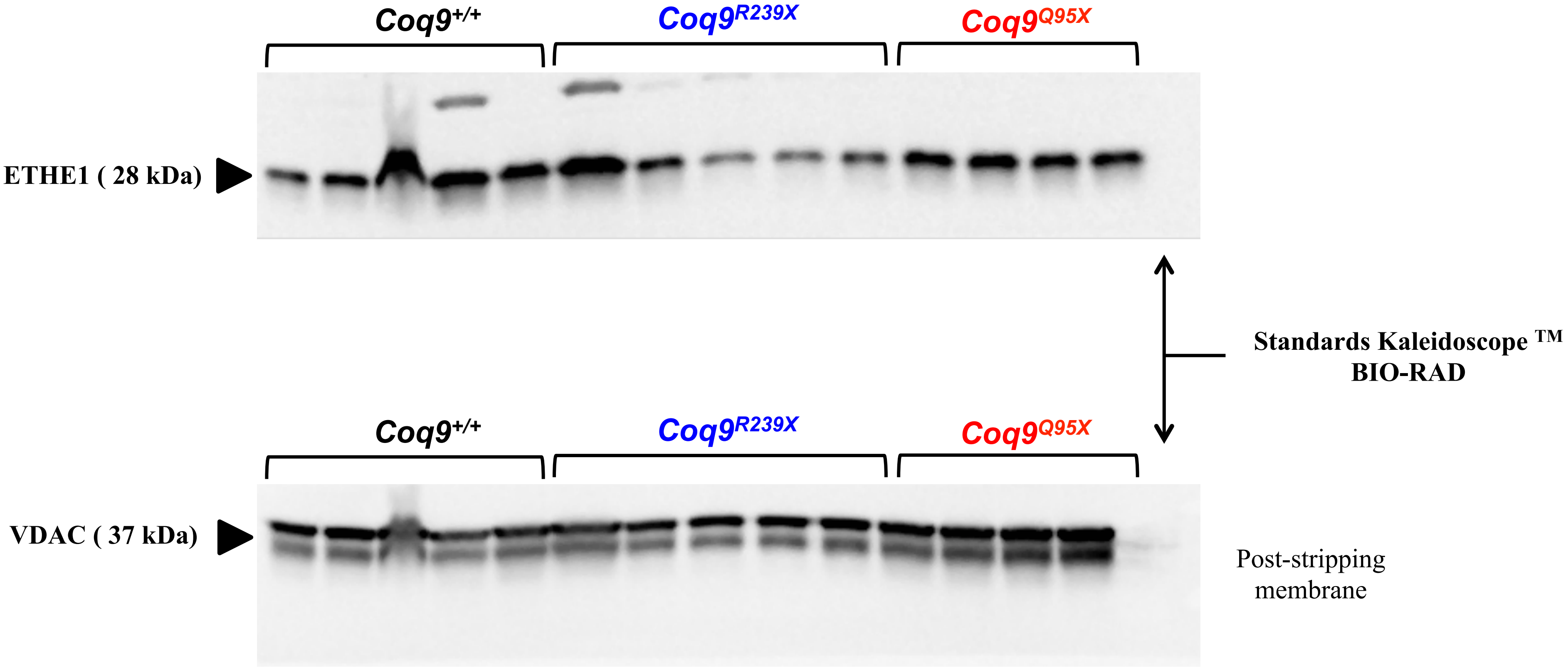

Note: lines 4, 5, 6, 7, 11 and 12 are represented in Figure 4G in the main text.

Figure 4H. ETHE1 in kidneys of wild-type and mutant mice.

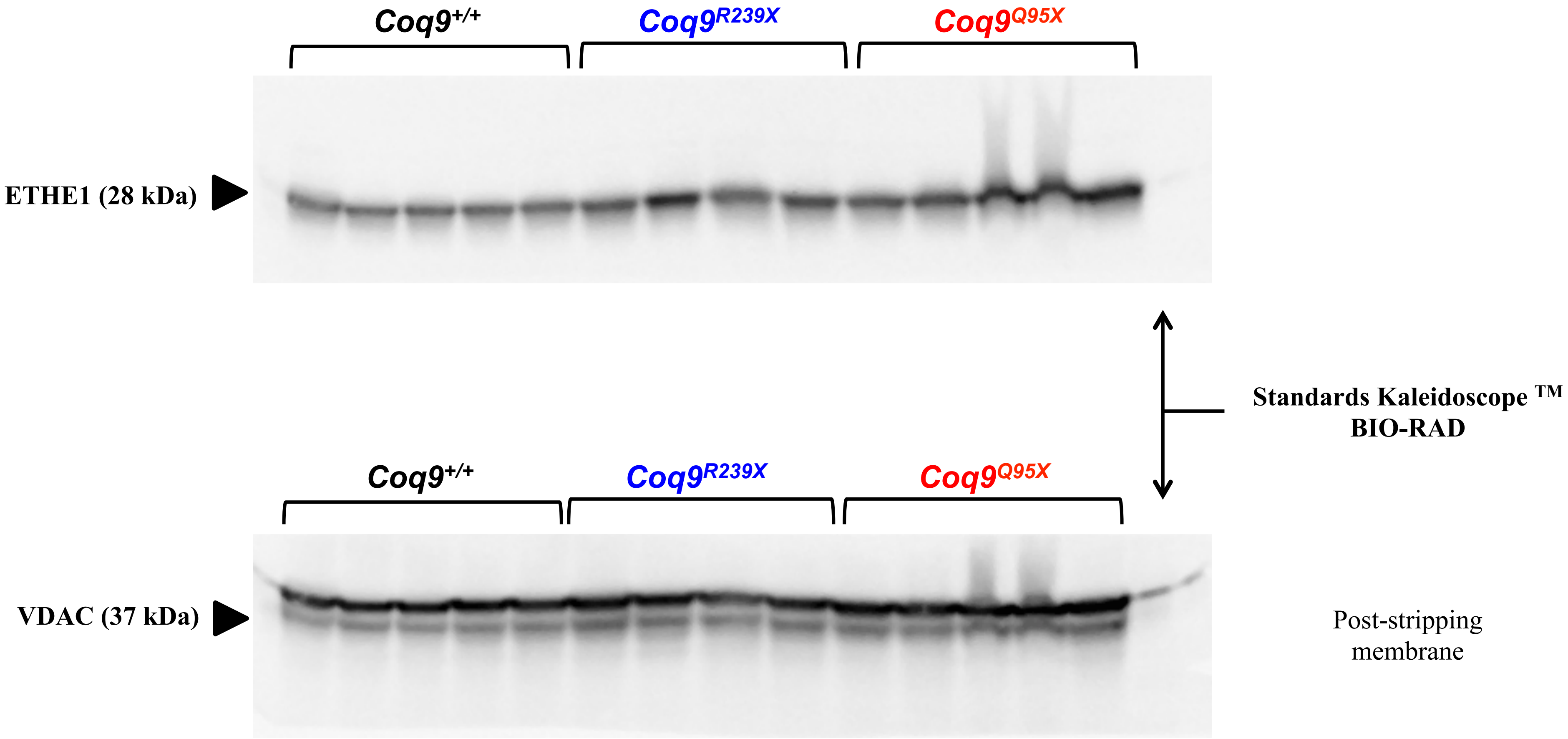

Note: lines 3, 4, 6, 7, 10 and 11 are represented in Figure 4H in the main text.

Figure 4I. ETHE1 in skeletal muscle of wild-type and mutant mice.

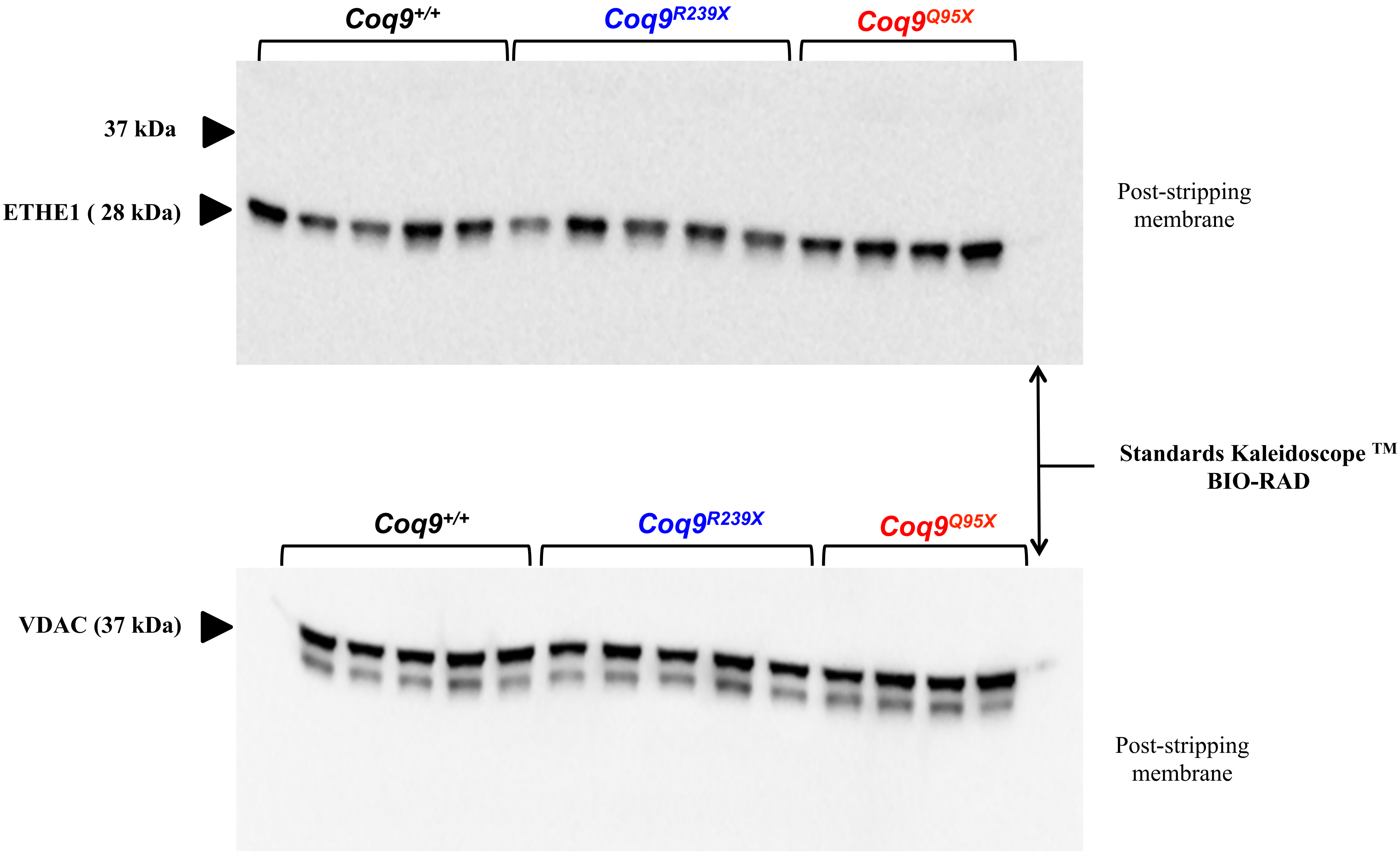

Note: lines 4, 5, 7, 8, 11 and 12 are represented in Figure 4I in the main text.

Figure 4J. SUOX in cerebrum of wild-type and mutant mice.

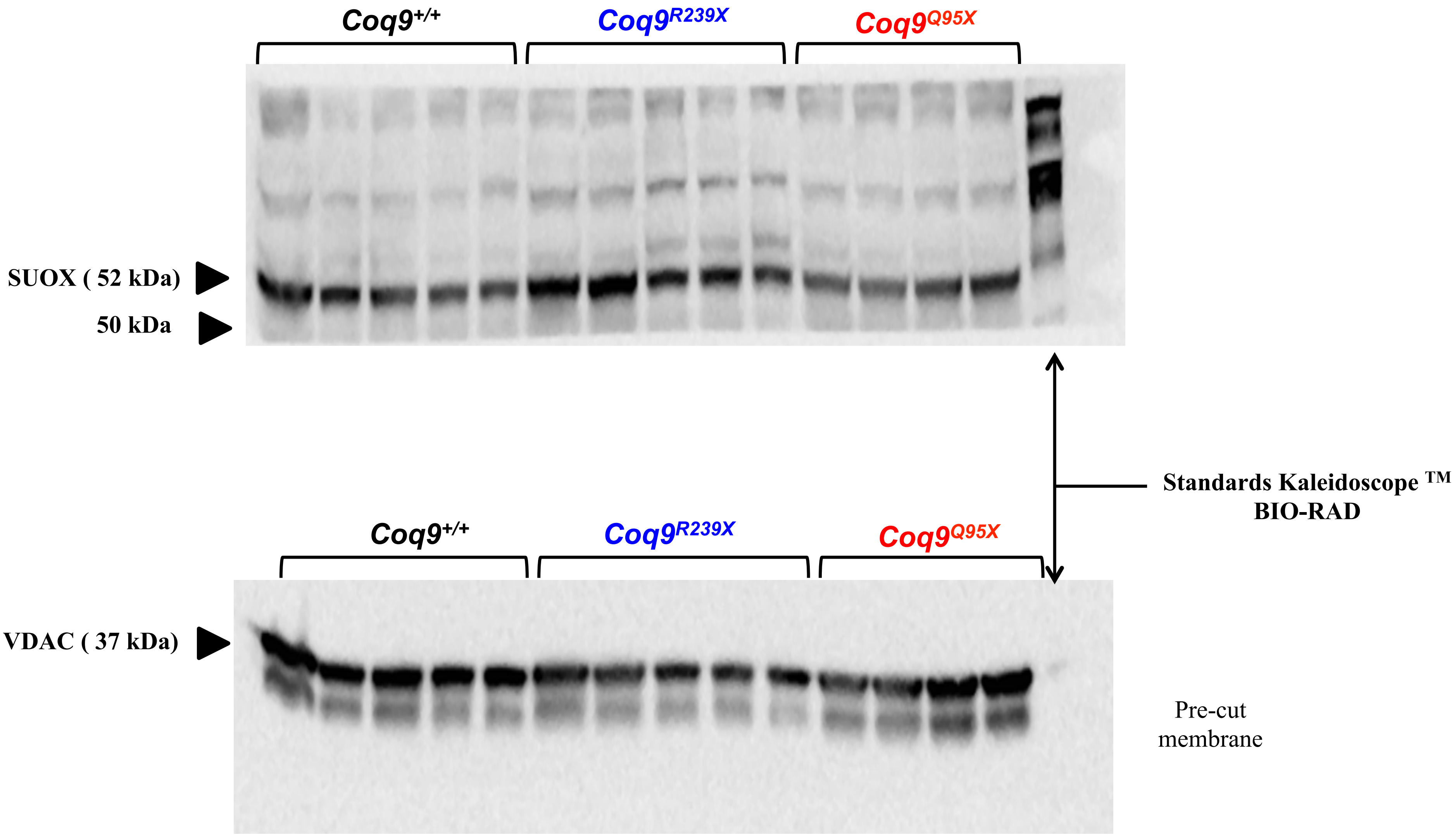

Note: lines 3, 4, 7, 8, 12 and 13 are represented in Figure 4J in the main text.

Figure 4K. SUOX in kidneys of wild-type and mutant mice.

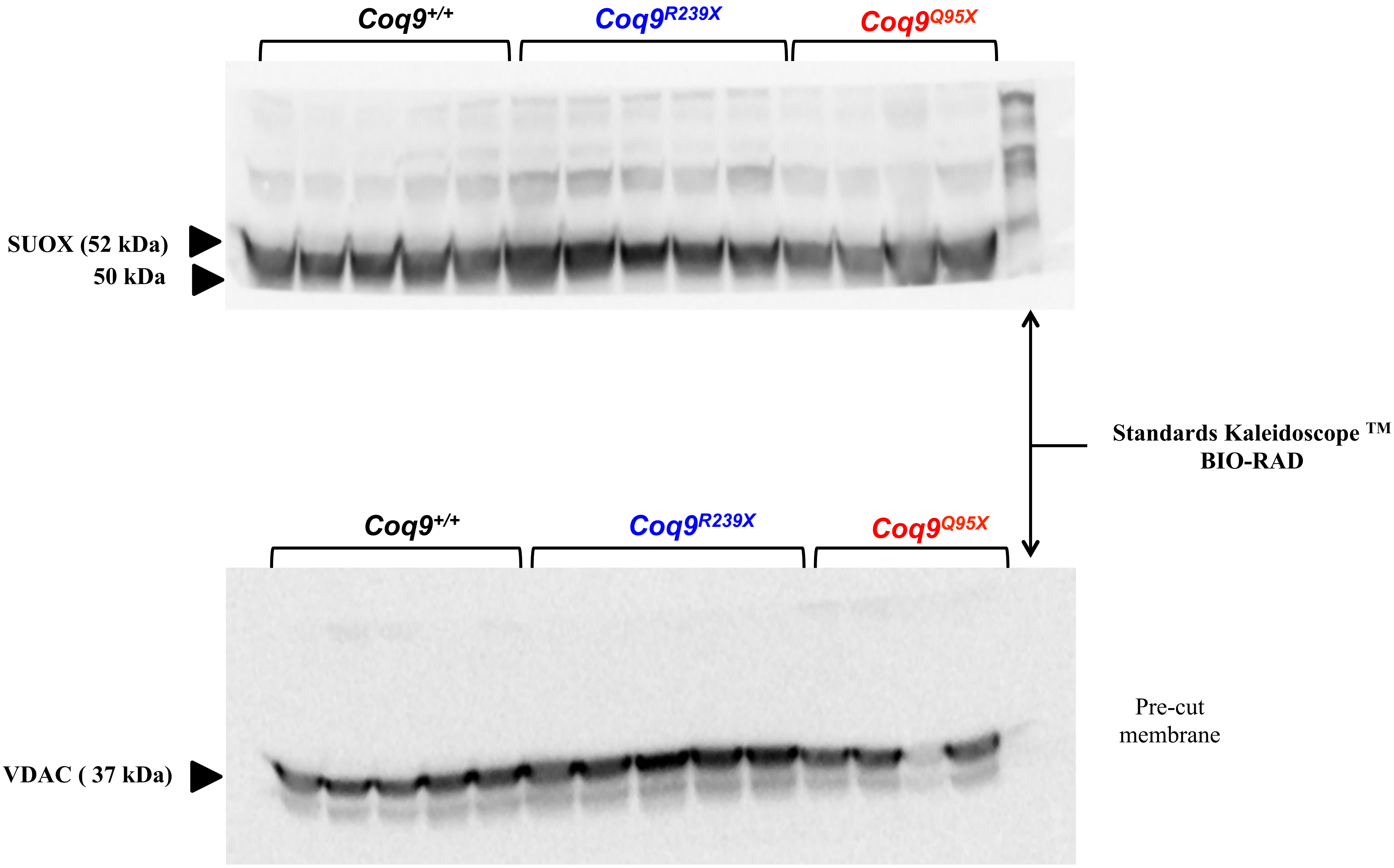

Note: lines 3, 4, 8, 9, 11 and 12 are represented in Figure 4K in the main text.

Figure 4L. SUOX in skeletal muscle of wild-type and mutant mice.

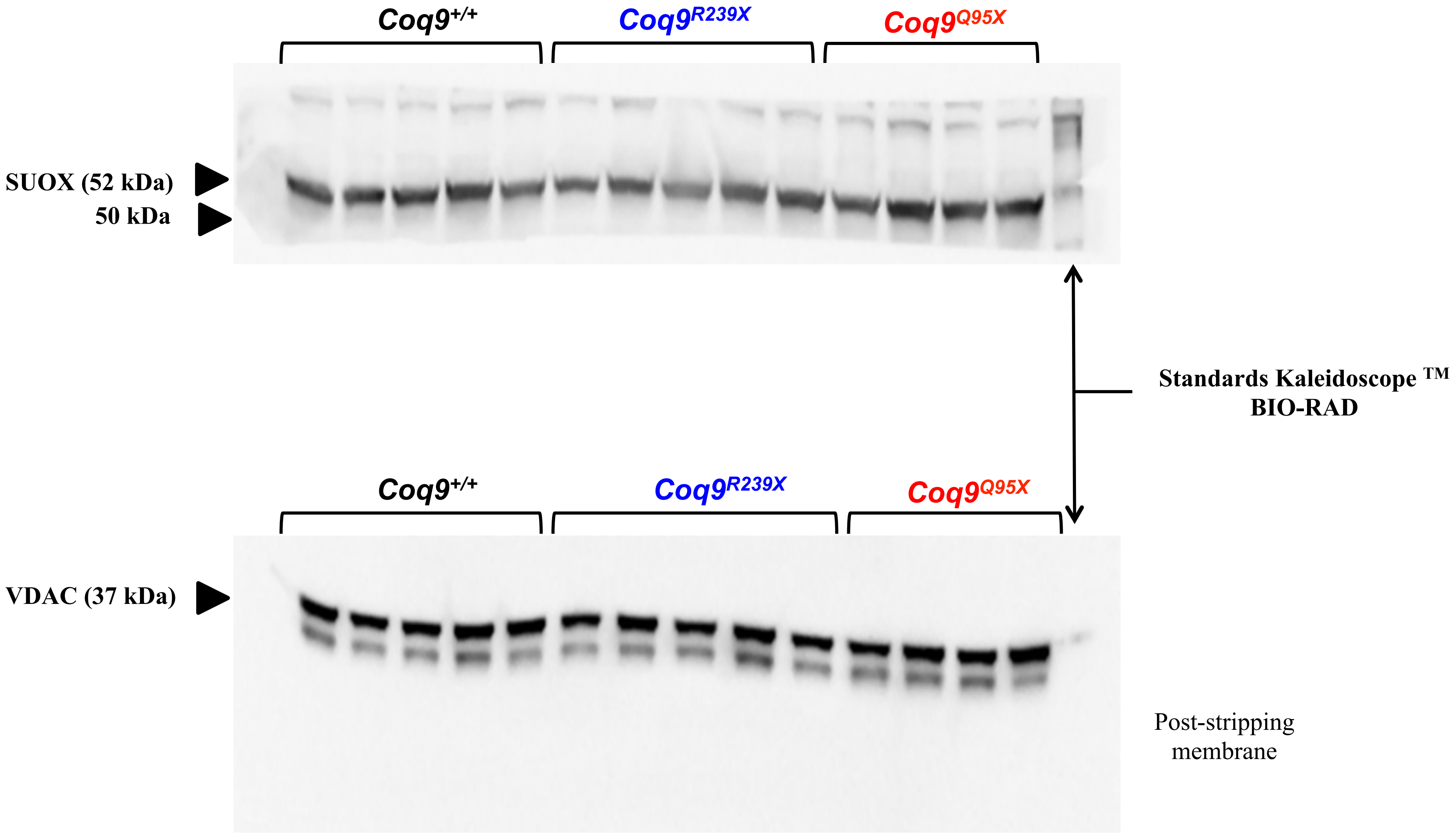

Note: lines 2, 3, 6, 7, 11 and 12 are represented in Figure 4L in the main text.
