# Supplementary material for: CoQ deficiency causes disruption of mitochondrial sulfide oxidation, a new pathomechanism associated with this syndrome
Source: EMBO Mol Med. 2016 Nov 17;9(1):78–95. doi: 10.15252/emmm.201606345 (PMC5210161; doi:10.15252/emmm.201606345)
Supplement: Supplementary file 7 — Source Data for Figure 6 [file EMMM-9-78-s006.pdf]

Figure 6C. GPx4 in cerebrum of wild-type and mutant mice.

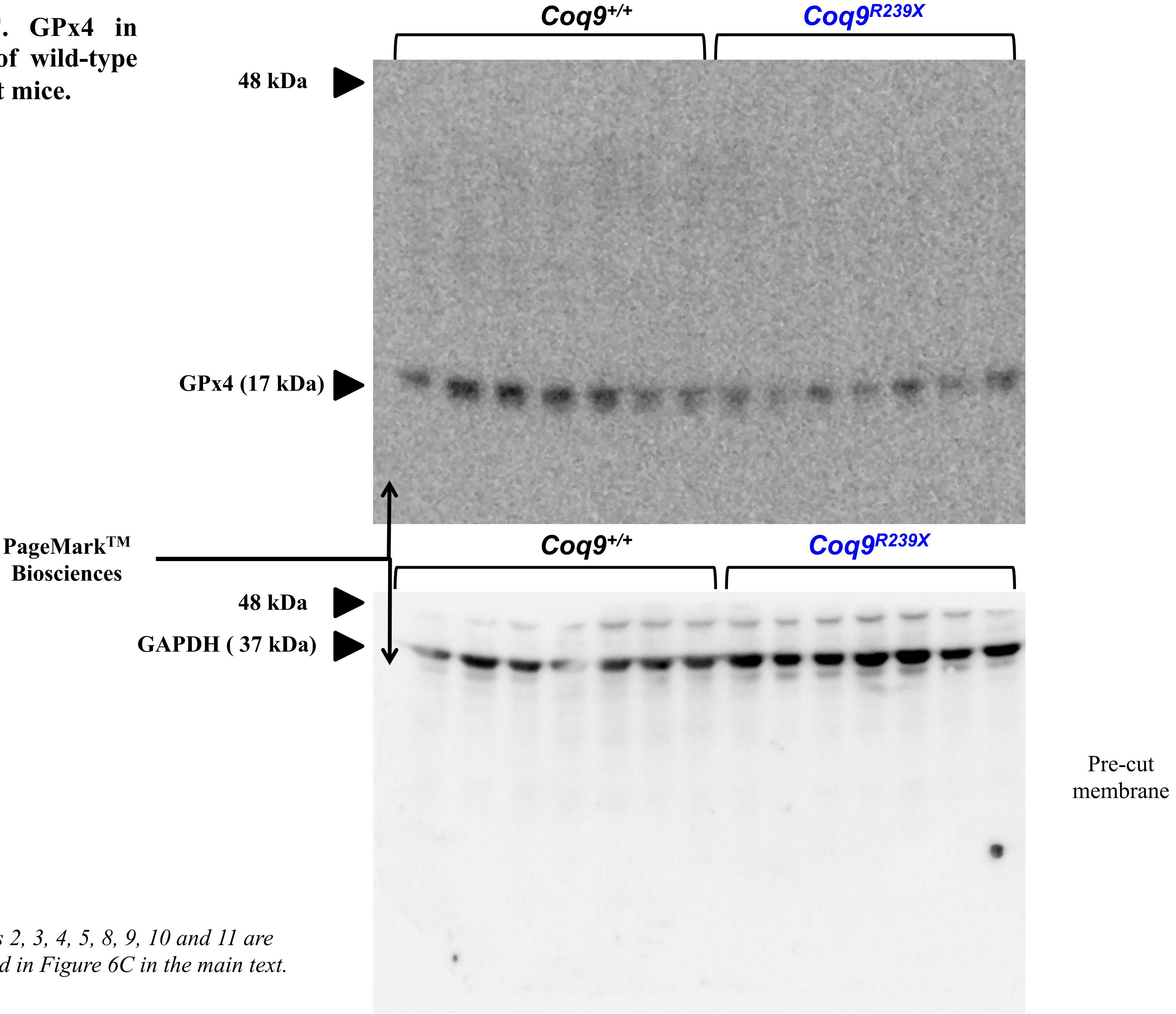

Note: lines 2, 3, 4, 5, 8, 9, 10 and 11 are represented in Figure 6C in the main text.

Figure 6D. GRd in cerebrum of wild-type and mutant mice.

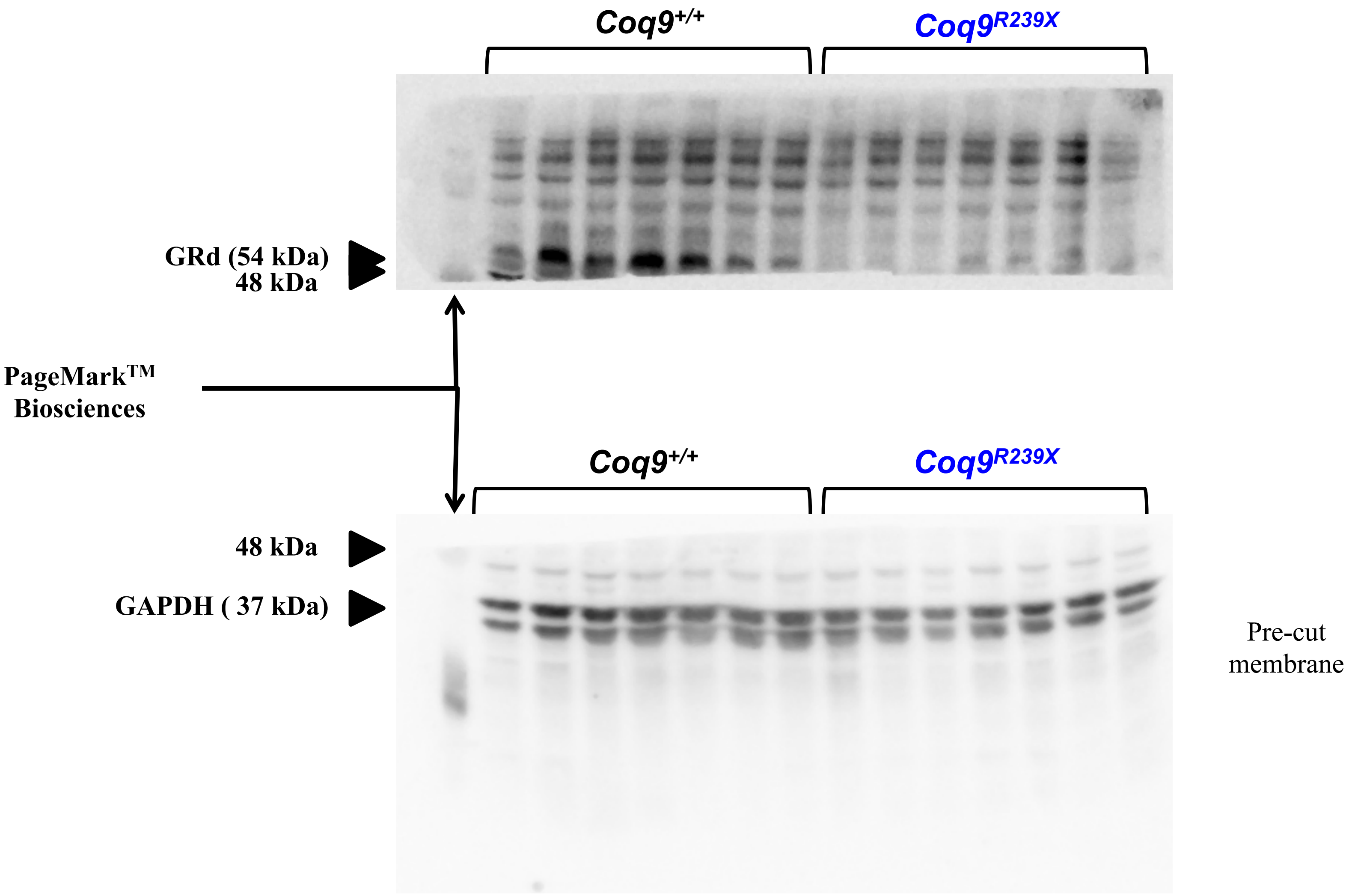

Note: lines 2, 3, 4, 5, 10, 11, 12 and 13 are represented in Figure 6D in the main text.
