# Supplementary material for: CoQ deficiency causes disruption of mitochondrial sulfide oxidation, a new pathomechanism associated with this syndrome
Source: EMBO Mol Med. 2016 Nov 17;9(1):78–95. doi: 10.15252/emmm.201606345 (PMC5210161; doi:10.15252/emmm.201606345)
Supplement: Supplementary file 8 — Source Data for Figure 7 [file EMMM-9-78-s007.pdf]

Figure 7A-B. SQR and TST in human fibroblasts after GYY4137 supplementation

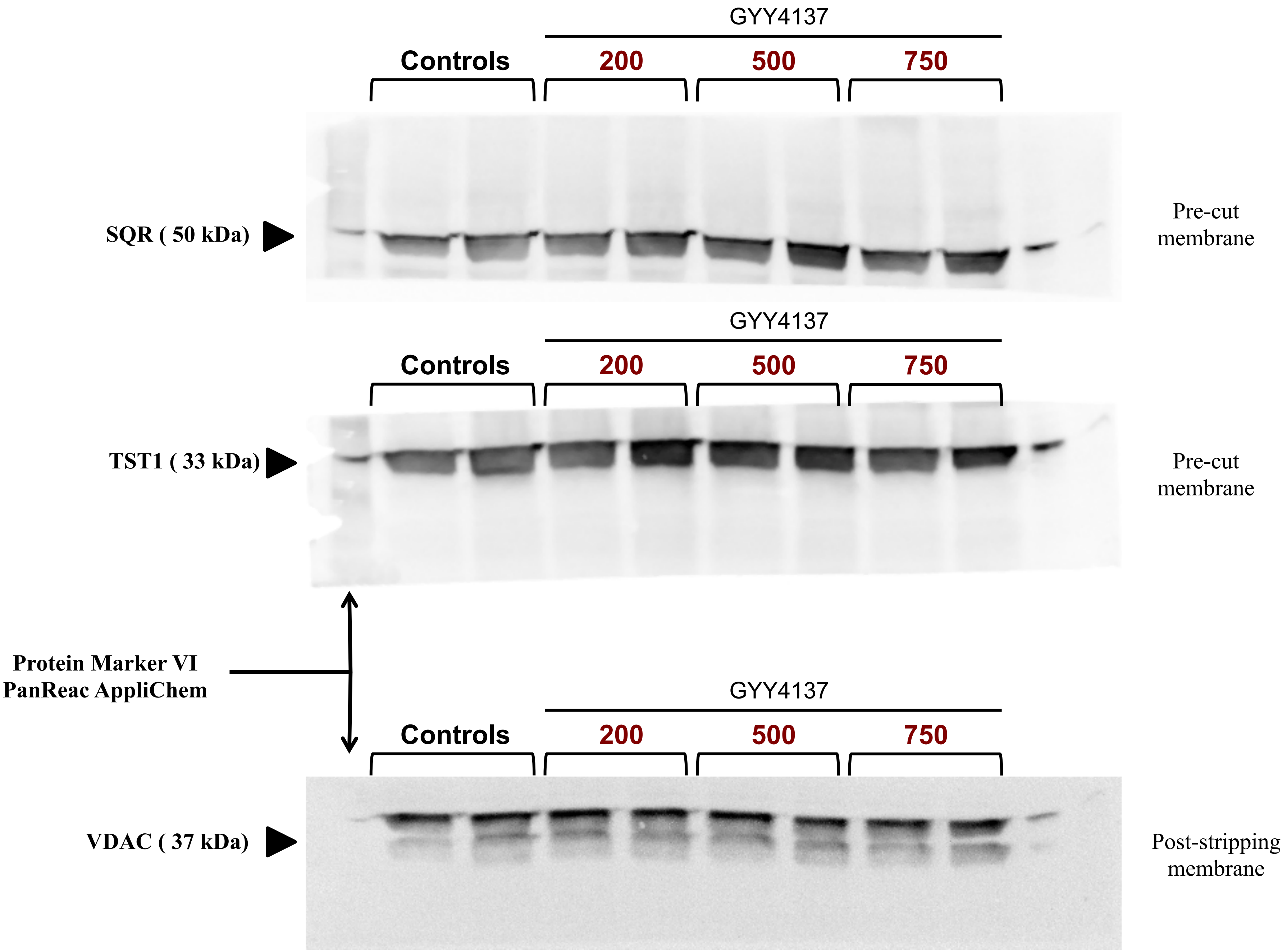

Figure 7C. TST in kidneys after GYY4137 supplementation

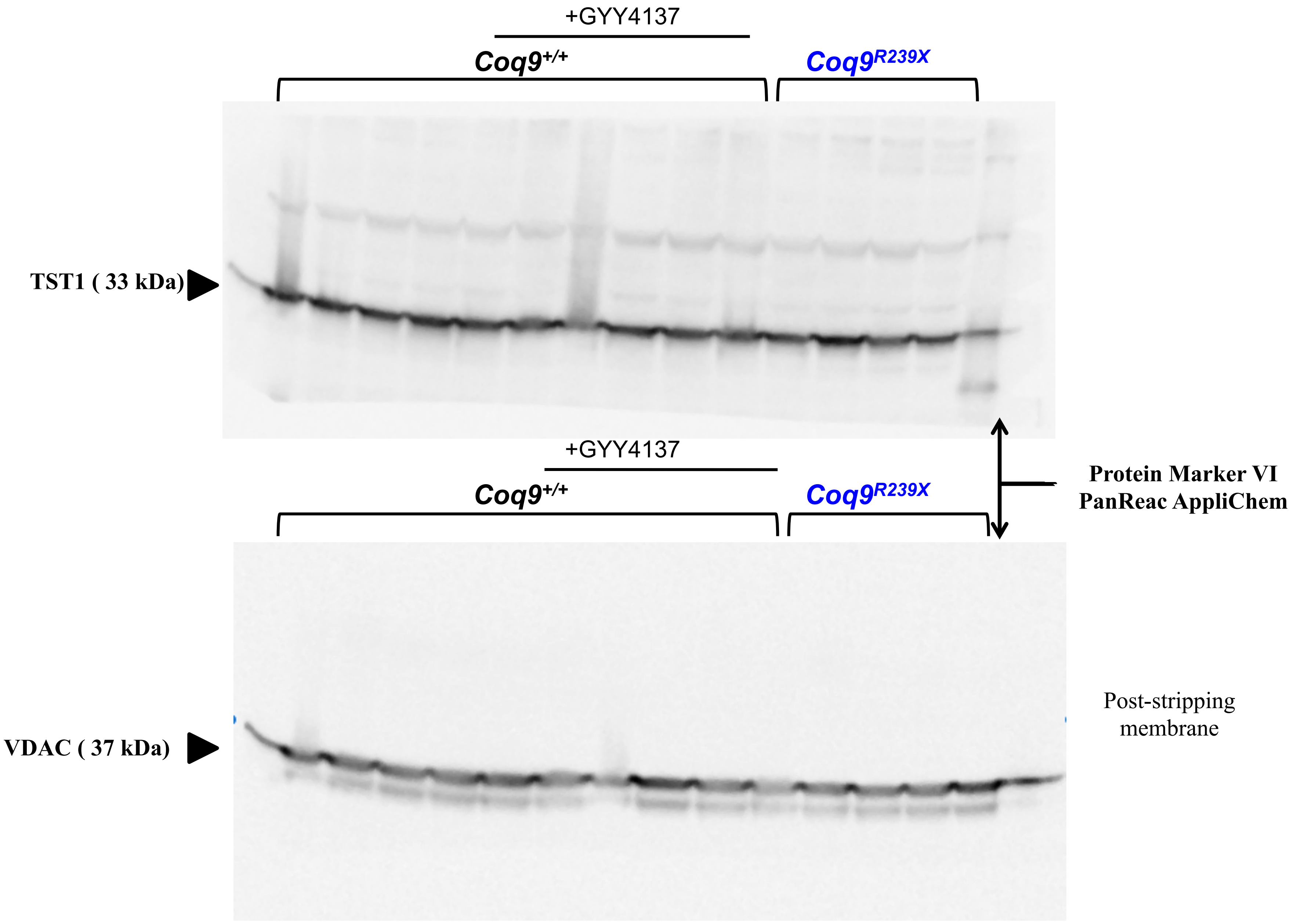

Note: lines 4, 5, 8 and 9 are represented in Figure 7C in the main text.

Figure 7D. TST in cerebrum after GYY4137 supplementation

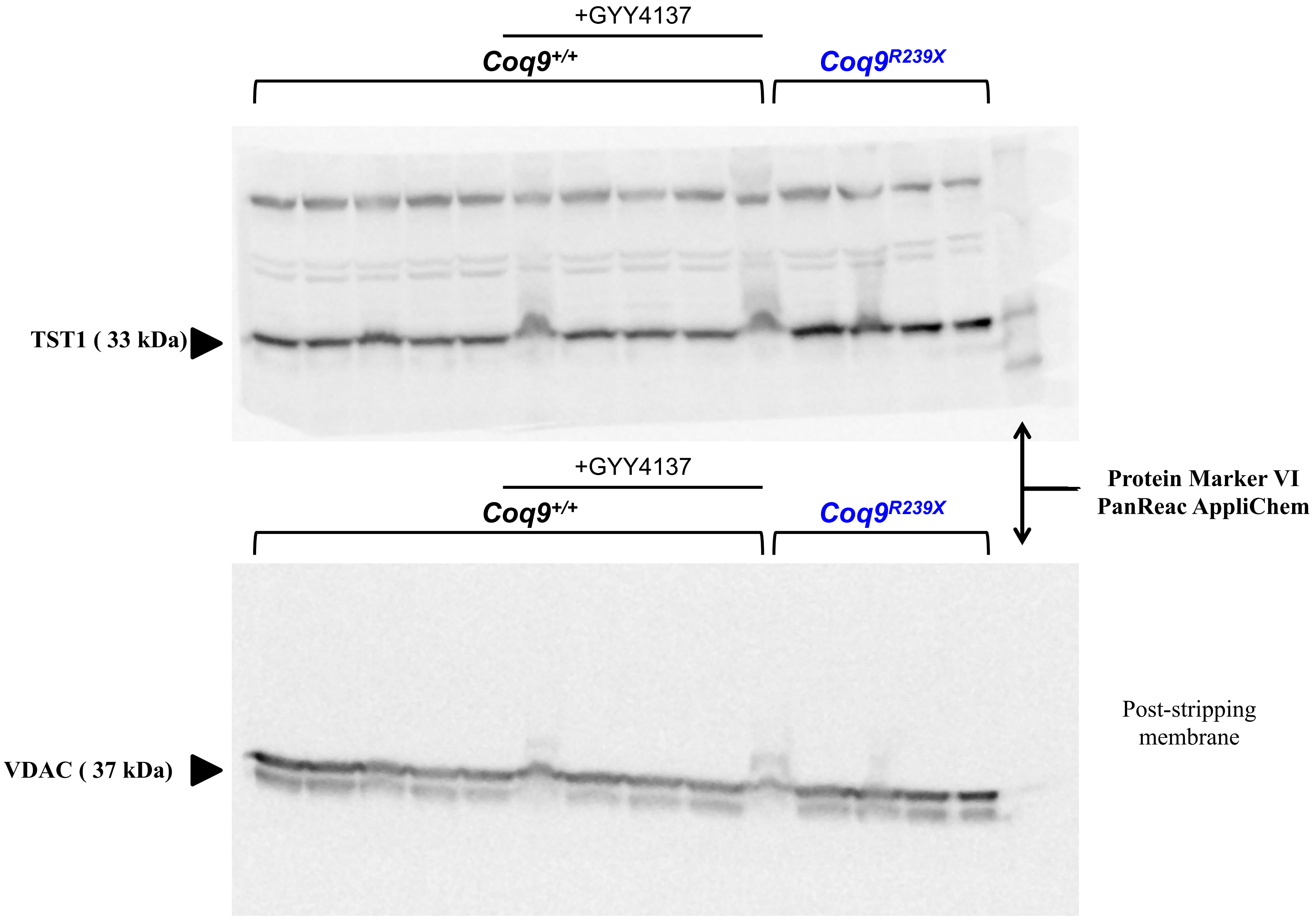

Note: lines 4, 5, 7 and 8 are represented in Figure 7D in the main text.
